# Investigation of serum protein profiles in scrapie infected sheep by means of SELDI-TOF-MS and multivariate data analysis

**DOI:** 10.1186/1756-0500-6-466

**Published:** 2013-11-14

**Authors:** Siv Meling, Olav M Kvalheim, Reidar Arneberg, Kjetil Bårdsen, Anne Hjelle, Martha J Ulvund

**Affiliations:** 1 Department of Production Animal Clinical Sciences, Section for Small Ruminant Research, Norwegian School of Veterinary Science, Kyrkjevegen 332-334, N-4325, Sandnes, Norway; 2 Department of Chemistry, University of Bergen, N-5007, Bergen, Norway; 3 Faculty of Health Studies, Sogn og Fjordane University College, N-6800, Førde, Norway; 4 Department of Innovation, Telemark Technological R&D Centre, Kjølnes ring 30, N-3918, Porsgrunn, Norway; 5 International Research Institute of Stavanger (IRIS, Biomiljø), P.O. Box 8046N-4068, Stavanger, Norway

**Keywords:** Scrapie, SELDI-TOF-MS, PCA, PLS-DA, Target projection, LC-MS/MS, Serum amyloid A, Sheep

## Abstract

**Background:**

Classical scrapie in sheep is a fatal neurodegenerative disease associated with the conversion PrP^C^ to PrP^Sc^. Much is known about genetic susceptibility, uptake and dissemination of PrP^Sc^ in the body, but many aspects of prion diseases are still unknown. Different proteomic techniques have been used during the last decade to investigate differences in protein profiles between affected animals and healthy controls. We have investigated the protein profiles in serum of sheep with scrapie and healthy controls by SELDI-TOF-MS and LC-MS/MS. Latent Variable methods such as Principal Component Analysis, Partial Least Squares-Discriminant Analysis and Target Projection methods were used to describe the MS data.

**Results:**

The serum proteomic profiles showed variable differences between the groups both throughout the incubation period and at the clinical end stage of scrapie. At the end stage, the target projection model separated the two groups with a sensitivity of 97.8%, and serum amyloid A was identified as one of the protein peaks that differed significantly between the groups.

**Conclusions:**

At the clinical end stage of classical scrapie, ten SELDI peaks significantly discriminated the scrapie group from the healthy controls. During the non-clinical incubation period, individual SELDI peaks were differently expressed between the groups at different time points. Investigations of differences in -omic profiles can contribute to new insights into the underlying disease processes and pathways, and advance our understanding of prion diseases, but comparison and validation across laboratories is difficult and challenging.

## Background

Prion diseases, like scrapie in sheep, are often called Transmissible Spongiform Encephalopathies (TSEs). These are fatal neurodegenerative diseases in a variety of host species, including humans. They are all associated with the conversion of the normal host cellular prion protein, PrP^C^, into the abnormal protease-resistant isoform, PrP^Sc^. The PrP genotype influences susceptibility, incubation period and clinical presentation, the V_136_R_154_Q_171_ allele being most highly associated with classical scrapie in sheep. To control and prevent spread of scrapie, genetic screening and breeding for resistance are widely used, and was implemented in the EU through Decision 2003/100/EC [1,2]. The PrP genotype is, however, neither a marker for definitive disease, nor the only genetic factor influencing prion diseases [3,4]. Despite the effort of reducing susceptibility, and monitoring and culling of ruminants, scrapie still exists [5,6].

As of today, much research into prion diseases has evolved around the prion protein itself through infection and dissemination studies, and relatively little has been done on other non-PrP^Sc^ disease processes. The most recent large scale survey on prevalent PrP^Sc^ in human appendix samples in Britain, suggests a higher prevalence of infection than formerly anticipated, in all human PrP genotypes, and these findings further necessitates focusing on various mechanisms in prion disease development and progression [7]. The variable incubation time, the complex epidemiology and different variables which may influence the clinical and pathological picture are increasingly important to elucidate [8-10]. Different -omic studies of tissues and body fluids, like serum, may potentially reveal markers that can contribute to unravel the intricate pathogenesis of prion diseases. Recently, several non-PrP^Sc^ proteins have been put forward as promising biomarkers for preclinical scrapie [11-15]. Identification of such non-PrP^Sc^ biomarkers may be crucial in future prion research.

The Surface Enhanced Laser Desorption/Ionization-Time of Flight-Mass Spectrometry (SELDI-TOF-MS) technology (Ciphergen Biosystems, Fremont, CA, USA) was designed to perform a mass spectrometry (MS) analysis of protein mixtures based on the mass-to-charge (*m/z*) ratio of the proteins, and on their binding affinity to the various chip surfaces. For a single charged protein, the molecular weight in Dalton (Da) usually corresponds well to the mass-to-charge (*m/z*) value, and the peak intensity corresponds well to the concentration in the sample. Different protein expression profiles may then be determined from these protein profiles by comparing the intensity of peaks of similar *m/z* value [16].

Proteins are good indicators of current cellular functions, and investigation into the serum proteome represents one direction in biomarker research [16]. One of the challenges in investigating the serum proteome is its complexity and the presence of high abundant blood proteins, particularly albumin. It is estimated that the high abundant proteins constitute 95% of the bulk mass of proteins, but they represent less than 0,1% of the total number of proteins [17]. These high abundant proteins may produce large signals and mask or interfere with the detection of other low abundant proteins [18]. To simplify the sample complexity, an up-front fractionation procedure is recommended in addition to the fractionation achieved by the chromatographic properties of the SELDI ProteinChip® Array technology [16,19,20].

Extracting crucial information from the retrieved mass spectrometry (MS) data can be challenging. These data often have a much higher number of variables compared to number of samples, they do not follow a normal distribution, there is heteroscedasticity and variables are highly correlated. For these reasons, much effort has been invested in finding reliable methods to assist the interpretation of such profiles. Machine learning methods represent one direction, and another is the latent variable (LV) approach where principal component analysis (PCA) is commonly used for unsupervised exploratory analysis of mass spectral data [21]. Partial least squares discriminant analysis (PLS-DA) is another method that utilizes the knowledge of group belonging to identify discriminating group data [22]. A problem with PLS-DA is that usually numerous latent variables are needed in order to achieve good discrimination between the groups and this can create interpretation problems. Following up with target projection (TP) method, the axis of best discrimination between groups can be achieved, and interpretation on a single predictive latent variable is obtained [23]. Rajalahti *et al.* developed a quantitative display called selectivity ratio (SR) plot for selecting biomarkers in spectral profiles. The SR plots provide both ranking and an objective measure of probability to guide the investigator in the selection process, resulting in a specific protein fingerprint profile that classifies unknown samples into controls or infected group [23,24]. It has been suggested that it is possible to classify samples based on multiple biomarker patterns, and therefore not constrained by the sensitivity and specificity of any single biomarker [16,20,25].

In this work, SELDI-TOF-MS technology was used in the analysis of pre-fractionated serum samples, and we describe the data processing steps and the following latent variable projection methods used to visualize the variation and highlight variables which separate the groups in question.

## Results

### Animals

**Figure 1:**
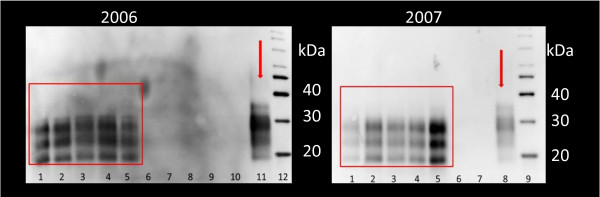
**Detection of PrP**^**Sc **^**by Western blotting.** WB using P4 antibody of homogenated brain material from animals and inoculation material used in this experiment. Lanes 1–5 (2006 and 2007) represent the scrapie inoculated animals. Lanes 6–10 (2006) and 6–7 (2007) represent the control animals. The lanes indicated by the arrow represent inoculation material used in the scrapie groups. Molecular markers were placed in lanes 12 and 9. PrP^Sc^ was detected in inoculation material and in all the animals from the scrapie groups.

At time of euthanasia, 23 weeks post inoculation (wpi), all the scrapie infected animals showed typical signs of scrapie, such as pruritus, ataxia, reduced live weight, weak coordination and poor wool quality. None of the animals in the control group showed any clinical signs of scrapie. Brain material from both groups and inoculation material used were examined by western blot (WB) for the presence of PrP^Sc^, and results are presented in Figure 1.

### SELDI-TOF-MS data processing and evaluation

Reproducibility of the SELDI-TOF MS analysis was evaluated on the basis of the calculated coefficient of variation (CV) of peak intensities and *m/z*. The pooled CVs (CVp) were calculated and results are in the same region as reported by others, and are shown in Table 1. CVp for mass accuracy across samples were all below 1%.

**Table 1:** Coefficient of variation for peak intensities across samples (ES data) and quality control (QC) sample (LS data)

**Sample ID**	**CVp%**
1	20.5
2	14.8
3	12.6
4	22.6
5	23.4
6	28.0
7	16.9
10	15.6
11	26.1
12	25.4
13	14.1
14	14.8
15	28.7
19	19.4
20	16.8
21	15.8
22	18.4
23	28.6
24	28.6
QC	28.2

Statistical description of the CVp calculated for each of the individual sample and QC sample. All the calculated CVp’s for peak intensities were below 28.7% which was in the same region as others have reported [19,25,28,29].

### Data analysis of clinical end stage data

**Figure 2:**
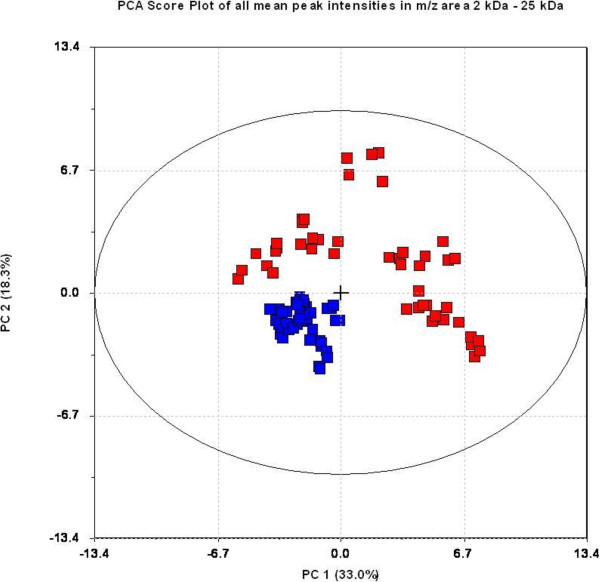
**Principle component analysis of 38 peak clusters from end stage study (ES) data.** Samples from scrapie affected animals are indicated in red, and healthy controls are indicated in blue. The first principal component explains 33% and second principal component explains 18.3% of total variation in data. Both these components visually separated the groups, and much of disease related variation contributed on first (PC 1) and second (PC2) principal components.

**Figure 3:**
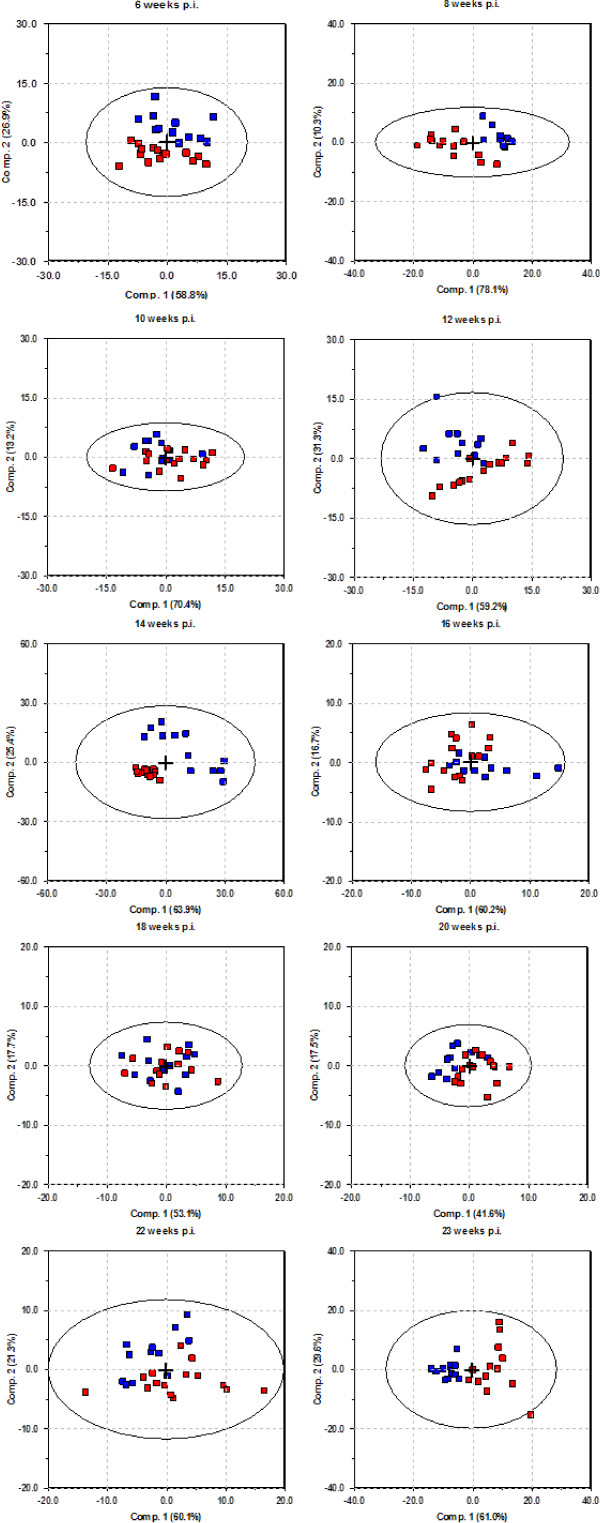
**Principle component analysis of longitudinal study (LS) data.** Samples from scrapie group are indicated in red, and samples from control group are indicated in blue. One PCA plot for each sample point; six, eight, ten, 12, 14, 16, 18, 20, 22 and 23 weeks of age/post infection.

PCA analysis was performed on MS data from both end-stage study (ES) and longitudinal study (LS) on the basis of peak clusters derived from biomarker wizard feature (BW) included in the Ciphergen ProteinChip® Software, and score plots are presented in Figures 2 and 3 respectively.

The PCA analysis was used solely for visualisation purpose. The score plots in Figure 3 demonstrated that the healthy animals and infected animals segregated well at the clinical end-stage (23 weeks p.i.), but poorly during the asymptomatic incubation period. Principal component one (PC 1) describes most of the variation in each data set, but how much of this variation is accounted for by scrapie is unknown, as this method does not take group belongings into account. Data sets from LS were not analysed any further with LV methods, due to the low number of peaks selected in BW, making these methods not suitable. The LS data was further analysed by the non-parametric Mann–Whitney U test for significant difference in individual peak intensity between the groups at each sampling time. The resulting peaks and their *m/z* value, significance level and fold change are listed in Table 2.

**Table 2:** Significant peaks in the longitudinal study and fold change

	**Weeks of age/post infection**									
***m/z***	**6**	**8**	**10**	**12**	**14**	**16**	**18**	**20**	**22**	**23**
**2030**						↓(1,9)**	↓(1,7)*			
**4395**					↓(5,9)***					
**4635**		↑(1,9)***				↓(1,3)**		↑(1,3)*	↑(1,4)**	↑(2,1)***
**5061**										↑(2,0)**
**5201**					↓(4,4)**					
**5695**	↓(2,4)***	↓(1,7)**		↓(1,5)*						
**5712**	↓(2,2)***	↓(1,7)**							↑(1,5)*	
**7542**					↓(3,3)***					
**8057**					↓(5,7)**					
**8509**					↑(3,8)**			↑(4,3)***		↑(3,1)***
**8625**				↓(1,8)*						
**8724**										↑(3,2)**
**8779**					↓(7,8)***					
**8796**		↓(1,9)**		↓(2,6)***	↓(4,4)***					
**8813**		↓(1,7)**	↓(1,2)*	↓(2,5)***	↓(3,2)***					
**9271**		↑(1,9)**			↓(1,7)*	↓(1,4)*		↑(1,3)**	↑(1,4)*	↑(2,4)***
**9478**		↑(2,0)***			↓(1,6)*	↓(1,3)**			↑(1,5)*	
**15073**		↑(27,2)***			↓(6,5)***					
**15278**					↓(6,0)***					
**16106**		↑(19,7)***			↓(5,9)**					

Peaks found to be significantly under- and overexpressed in the scrapie group. Arrow indicates change in scrapie group relative to control group at different time points post infection (weeks of age/post infection). ↓ - under-expression, ↑ - over-expression, *m/z* – mass in Dalton, average fold change in expression is given in brackets. * = p < 0.05, ** = p < 0.01, *** = p < 0.001.

**Figure 4:**
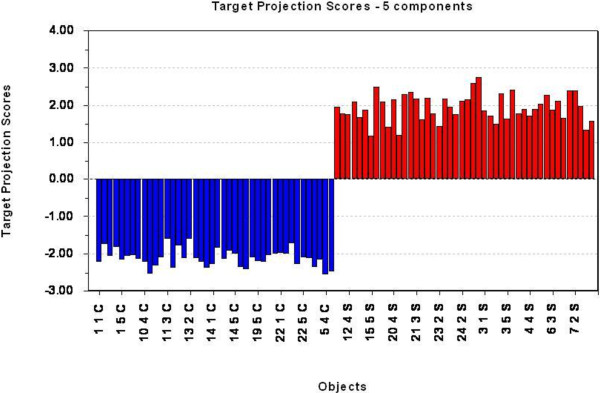
**Target Projection score indicating grouping for each sample.** All the scrapie samples (red) have a positive score value, and all the samples from healthy controls (blue) have a negative score value. The samples are indicated on the x-axis, and the target projection score on the y-axis. The TP model was able to separate the two groups with no misclassifications.

**Figure 5:**
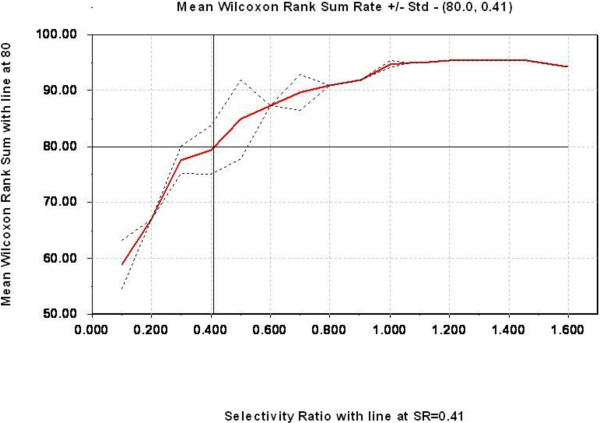
**DIVA plot.** A DIVA plot of the TP model with the red solid line indicating mean Wilcoxon classification rate and standard deviation (dashed line), and SR values on the x-axis. Horizontal line indicates the chosen 80% MWCR, and the vertical line indicating the resultant SR threshold of 0.41.

**Figure 6:**
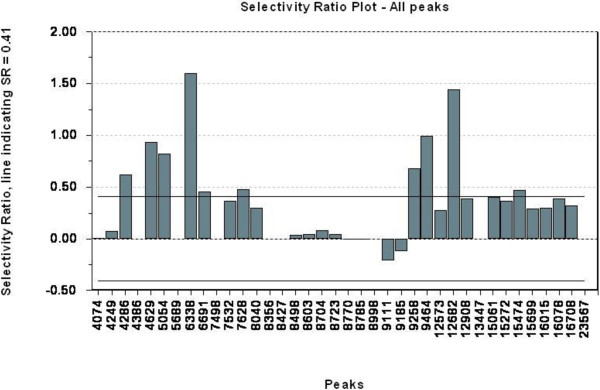
**Selectivity Ratio plot for all peaks in the model.** A bar chart of all the peaks (x-axis) used in the model and their calculated SR value (y-axis). The two horizontal lines indicate the SR threshold with an absolute value of 0.41. Ten peaks have a SR value above this threshold.

**Figure 7:**
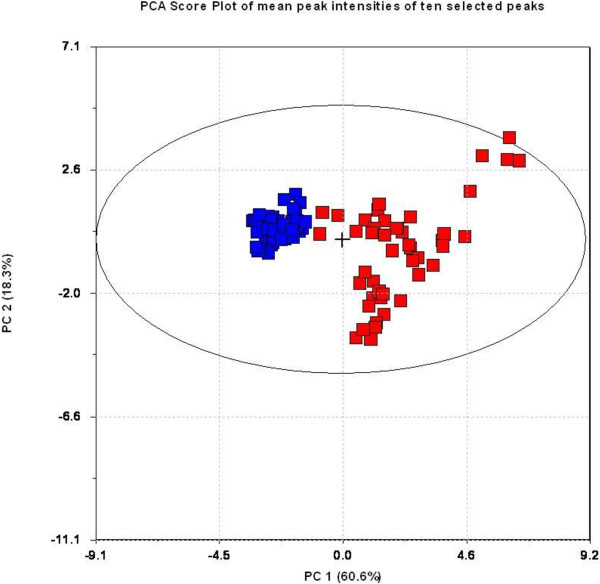
**PCA plot of distribution of the two groups using only ten peaks.** The ten peaks selected from the analysis were used in a PCA plot and there was good visual separation of the groups with PC1 accounting for about 60% of the variation in the dataset based on these ten peaks.

**Figure 8:**
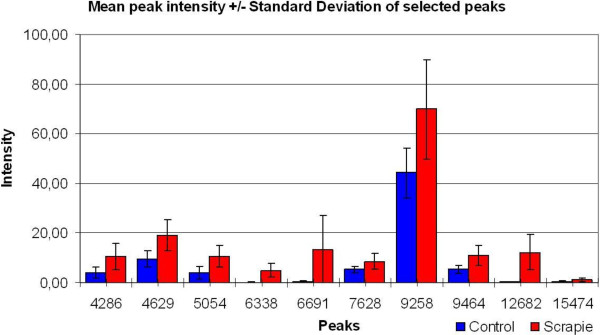
**The mean peak intensity and standard deviation for the ten selected peaks.** A bar chart of the ten selectee peaks, with *m/z* on the x-axis and intensity on y-axis. Scrapie samples were indicated in red, and controls were blue. Standard deviation for each mean was indicated on each of the bars.

Only data from clinical end stage study was further analysed by PLS-DA using group classification as the dependent variable. Five (5) components were shown to possess predictive information according to cross validation. This model used 70.6% of the variables in the protein profile (explanatory variables) and explained 97.8% of the variance in group membership (response variable), indicating an excellent predictive model. This PLS-DA model was used as the basis for the TP model and the resulting TP scores are graphically presented in Figure 4, showing excellent discrimination between healthy controls and infected animals. The TP model uses only 19.7% of the variables in protein profiles to explain the same 97.8% of the variance in the group membership. This indicates that most of the variation in the mass spectral data was not related to the disease status, and therefore removed in the TP model. The two models are summarized in Table 3. By choosing 80% mean correct classification rate (MCCR) for the Mean Wilcoxon Rank Sum as the sensitivity threshold for selecting discriminating peaks, the Discriminating Variable (DIVA) plot indicated the corresponding Selectivity Ration (SR) threshold to be 0.41 (Figure 5). From this we were able to select ten variables, presented in the Selectivity Ratio Plot in Figure 6, with individual Wilcoxon classification rate (sensitivity) in the range of 82 – 95 per cent (Table 4). These ten peaks were used in a new PCA analysis for a visual impression of the distribution of animals on the basis of these ten peaks, Figure 7. As illustrated in this PCA Score Plot, the two groups were well separated along PC 1 which indicated that these ten variables were highly related to group differences, i.e. scrapie *versus* healthy. The intensity and standard deviation of each of these SELDI peaks represented by *m/z* value were plotted in a bar diagram and presented in Figure 8. From this we can see that all of these ten proteins were over-expressed at the clinical end stage of scrapie.

**Table 3:** Modelling results of both PLS-DA and TP predictive models before and after peak selection

**Data**	**No. of spectra**	**No. of PLS comp**	**R2 (XPLS-DA)%**	**R2 (XTP)%**	**R2(y)%**	**% MCCR (DIVA)**	**SR limit**	**No. of selected peaks**
C/S	88	5	70.6	19.7	97.8	80	0.41	10 (26%)
C/S^a^	88	4	91.1	48.6	87.8			

No. of spectra: 19 individuals in 3–5 replicates; R2 (XPLS-DA): 70.6% of total variance in X is explained in the PLS-DA model; R2 (XTP): 19.7% of total variance in X is explained in the TP model; R2(y): 97.8% of total variance in the response variable. y. is explained in both models; MCCR: mean correct classification rate; No. of selected peaks with percentage of total peak selection in brackets; C/S^a^: modelling results after reduction of subset to only include the selected peaks.

**Table 4:** Selectivity ratio value, Wilcoxon classification rate and univariate p-value for each of the selected variable

**Variable ( *m/z *)**	**SR**	**% Wilcoxon Classification Rate**	**Mann–Whitney U test p-value**
4286	0.62	92	0.00E + 00
4629	0.94	94	0.00E + 00
5054	0.82	92	0.00E + 00
6338	1.60	94	0.00E + 00
6691	0.46	95	0.00E + 00
7628	0.48	84	3.87E-08
9258	0.68	87	1.50E-09
9464	0.99	95	0.00E + 00
12682	1.44	95	0.00E + 00
15474	0.46	82	1.78E-07

### Protein identification

**Figure 9:**
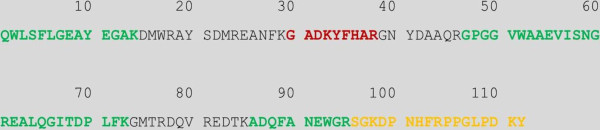
**SAA sequence and the identified peptides.** Here is the total ovine Serum Amyloid A protein sequence and peptides identified by LC-MS/MS are highlighted. The peptide in red was identified with low confidence, the peptide in yellow was identified with medium confidence, and the peptides in green were identified with high confidence.

Serum Amyloid A (SAA) protein (gi1173354) was identified by eight peptides using high confidence filter, giving coverage of 45.54%, and SAA was only identified in the scrapie sample. The peptide sequence of SAA and the identified peptides are shown in Figure 9. SAA consists of 112 amino acids and has a theoretical molecular weight of 12 688 Da which corresponded well with one of the selected SELDI peaks with an *m/z* of 12 682. The data of this SELDI peak are presented in Table 5.

**Table 5:** **Results from data analysis for SELDI peak with *m/z *** **of 12682**

**Peak *m/z***	**SR**	**Mean Intensity – Control**	**Standard Deviation – Control**	**Mean Intensity – Scrapie**	**Standard Deviation – Scrapie**	**% Wilcoxon Classification Rate**	**Mann–Whitney U test p-value**
12682	1.44	0.24	0.16	12.20	7.21	95	0.00E + 00

## Discussion

In this study, we have evaluated the use of SELDI-MS-TOF data and latent variable methods to create and analyse serum protein profile data to discriminate healthy sheep from sheep with scrapie at various stages during the incubation period and at the clinical end-stage. Batxelli-Molina *et al.* discriminated sheep with early phase scrapie and healthy controls by the use of four SELDI peaks with sensitivity and specificity of 87.3% and 88.1%, respectively [11]. We were able to create a good predictive regression model only from the clinical end stage data, and based on ten peaks, to discriminate scrapie affected animals from controls with a sensitivity of 87.8%. One of these ten selected SELDI peaks had a relatively high intensity in the scrapie group and was barely detectable in the control group. This peak had a mass (*m/z*) of 12 682 Da and a mean sensitivity of 95%. Based on results from LS-MS/MS analysis of samples from both control groups and scrapie groups, this peak was identified as serum amyloid A (SAA). The finding corresponds well with our previously published data on quantitative measurement of SAA in serum samples from these animals [26].

A range of different univariate and multivariate data analysis methods and different software have been used for analysing SELDI spectral data [11,16,17,19,25,27-30]. We believe that multivariate methods based on latent variables are better suited, as these methods can handle data with more variables than observations and data which are noisy and highly collinear [22,31,32]. They provide a good tool for visualization of the data, detection of patterns and object classification. Latent variable models reduce dimensionality of the data and reveal the underlying concept and structure in them. These methods have been reported by others to produce good results from SELDI-TOF MS data [27]. However, due to the few peaks (variables) in datasets from the longitudinal study, we were not able to create a predictive model without increasing risk of over-fitting the regression model. We were not able to define valid components in the PLS-DA model and at the same time achieve satisfactory cross validation of data. Results from the longitudinal study were therefore only evaluated visually by the PCA method, and individual peaks were evaluated for significance through Mann Whitney U test. Although significant p-values were observed at each sampling time, these results should be interpreted with care due to poor reproducibility of the SELDI-TOF-MS analysis and the risk of false positives due to the “multiple comparisons problem” arising when a high number of peaks are independently compared between the two groups. PCA is a powerful technique for data visualization, but it is an unsupervised method including all variance in the data into the analysis, and does not use any a priori information regarding group membership [32]. Much of this variance may also be due to other non-scrapie related differences between the animals such as sex, age, genetics, sampling time and individual physiological factors. Important biomarker patterns in serum proteome may be buried under such major differences and by using methods taking group membership into account, disease relevant differences may become clear. We have illustrated this by using PLS-DA to analyse ES data, where the model focuses on maximum separation of the two groups, in contrast to maximum variation in the PCA model [22,33]. PLS-DA model gives rise to large numbers of PLS components required to describe the majority of the variation in the data, and by combining these PLS components into a single TP component, which represents the direction in the multivariate predictive space with strongest relation to the response, interpretation becomes easy [34,35]. The information with no correlation to group membership has then been removed, and the TP score vector displays the discriminative information between the two groups on a single scale. This is illustrated and summarised in Table 3, where we show that total variance in data used to describe the predictive model was reduced to 19.7% in the TP model, from 70.6% in the PLS-DA model. The TP model also provides a quantitative measure of each original variable’s contribution to the discrimination between groups, but as peaks with large variance and little correlation to group membership may dominate over peaks with little variance and high correlation to group membership, this could not directly be used to select interesting peaks [34]. The selectivity ratio (SR) for each variable on the TP component is directly related to each variable’s ability to predict group membership and this was used to select variables in the model [23,24].

As described by Rajalahti *et al.*, a sensitivity level, or correct classification rate, for a set of peaks can be chosen individually for each data set and this is done statistically by the non-parametric Wilcoxon Rank Sum test. Completely random classification with equal number of samples in each group then gives a correct classification rate of 50%, and correct classification of all the samples will have a CR of 100% [23,24]. Setting the sensitivity threshold must balance the risk between selecting false biomarkers and missing important ones. In this study, we chose a mean sensitivity level/correct classification rate of 80% for the selected variables which gave a selectivity ratio (SR) value of 0.41, this is illustrated in the DIVA plot in Figure 5. Further on, this SR value was applied to all the variables in the Selectivity Ratio plot, Figure 6, and ten SELDI peaks qualified for selection by having a SR value above this threshold.

For two-group comparisons, like in this work, receiver operating characteristics (ROC) curves could be used to compare the sensitivity and specificity of a biomarker candidate at different cut-off values for peak intensity [36]. But as correct classification rate is identical to the sensitivity in a binary classification it will give us the same picture, only that the DIVA plot expands into the multivariate space.

The ten selected SELDI peaks were used in a PCA plot in Figure 7 to illustrate how well they separated the two groups in question along the PC 1. Figure 8 illustrates the intensity of these ten peaks in the SELDI spectra, and the increased expression in the scrapie group compared to the control group is probably related to the clinical status of the animals.

One of these peaks, with the *m/z* of 12682 Da, was identified by LC-MS/MS as serum amyloid A (SAA), which is a major acute phase protein (APP) in sheep. It has been quite common to identify acute phase proteins as discriminating biomarkers between groups of affected and not affected individuals, as these are highly sensitive reactants produced in response to an insult [18]. They are, however, not very specific, although different insults may produce different patterns of acute phase response (APR). Many of the reported diagnostic SELDI peaks have been found to be acute phase proteins, and are described in several reviews [11,19,37,38]. SAA is primarily induced by pro-inflammatory cytokines such as IL-1β, TNF-α and IL-6, which are released by a variety of cells including activated tissue macrophages and blood monocytes in response to injury [39,40]. Sheep with natural scrapie, and mice with experimental scrapie, show reactive astrocytosis and microglia activation and increased cytokine expression in the brain at the time of clinical signs and neuropathological changes [41-43]. These cytokines can cross over into the blood and initiate a systemic APR with increased synthesis of APPs from hepatocytes, such as SAA [44]. Coe et al. reported an increased level of serum amyloid P in plasma of mice with scrapie as evidence for systemic inflammatory response to scrapie [45]. Batxelli-Molina *et al.* identified transthyretin as being under-expressed in sheep with clinical scrapie [11]. Transthyretin is a negative APP expressed at lower levels during an APR along with the other negative APPs. Although identification of APPs as biomarkers of disease has not been considered significant, we believe that identification of any protein, regardless of specificity that significantly differs between scrapie affected and healthy controls, will contribute to novel information of underlying pathological processes of scrapie. The long incubation period, large variety in clinical presentation, as well as lack of direct link between neuropathology, PrP^Sc^ dissemination and clinical presentation, create the need for new knowledge of underlying processes at all stages of scrapie. Identification of discriminating proteins will contribute in this matter.

The SELDI-TOF-MS may be an excellent tool for protein profiling due to its high throughput, but, as this work has shown, there are too many technical limitations resulting in lack of peak identification and poor reproducibility to make this the technique of choice in the search for specific biomarkers. The challenges and limitations associated with SELDI-TOF-MS are nicely reflected by the poor reproducibility between our longitudinal and end point studies, and the low number of peaks detected at some time points, like 10 and 18 weeks. The method failed to detect the peak with *m/z* 12 kDa at both ES and LS, even though this peak separated the groups well and had high intensity in the ES study. Even though there are a number of peaks found to be significantly under- and overexpressed in the scrapie group compared to the control group in the LS data, the findings are of limited value, as long as the peaks are not identified as specific proteins which can elucidate specific pathological pathways of processes. It is also uncertain whether these individual peaks are separate proteins, several peaks can represent the same protein with different charges or modifications. We also noticed that there were large differences between the different time points, even though all the samples included in the LS were run randomly at the same time. This could be due to introduction of variables during handling and pre-processing of samples, especially from the initial fractionation step. The difference in number of peaks detected in each group could be due to suspected variation in quality and quantity in the FT fraction. As pointed out also by Van Gorp *et al.*, many promising studies on discriminating SELDI peaks have been published, but few follow-up papers on peak identification and validation have been published [46]. Barr *et al.* actually proposed a protein fingerprint for TSE infection in blood [47].

To create a proteomic profile able to detect sheep infected with scrapie during the incubation period with high sensitivity and specificity, rigorous testing of a large number of animals would be necessary, in addition to eliminating variability through sample handling and analytical procedures. In addition to scrapie, other neurological diseases would have to be similarly mapped. The reproducibility and validity of discriminating proteomic profiles would need to be confirmed across different laboratories and animal groups, including different genotypes, scrapie strains and age groups. One of the major limiting factors of SELDI proteomic profiles is the lack of direct comparisons of SELDI peaks based solely on *m/z*. Differences in experimental set-up from animal model to data analysis result in poor reproducibility in number of peaks detected, peak height and *m/z*, making the resultant peak list incomparable [48]. Comparison of SELDI data from different sample sets, different runs on the same or across SELDI-TOF-MS instrument(s) have resulted in considerable variation in number of discriminating peaks [37]. Comparisons made across different studies may also be misleading, as one protein species can generate about ten major peaks and many minor satellite peaks due to chemical reactions that may take place during the sample preparation and analysis. Proteins with approximately the same mass will show up with overlapping peaks, and spectra obtained with different machine settings can look different [49]. Our results also confirm this problem, as the samples set for LS and ES were prepared and analysed on two different occasions, and we were not able to reproduce the exact same results in the end point data sets. The relatively high CVp seen for peak intensity both within and between runs, indicate that slight changes in peak intensity between groups may not indicate an actual difference between groups, and thus careful interpretation of results was necessary. This problem may be overcome by considerably increasing the number of animals in each group. Results across different age-groups were not compared, as natural changes in protein profiles related to age changes may overshadow the difference due to disease status. We worked with very similar groups to enhance differences relating to scrapie, and minimize differences related to pre-analytical factors like age, sex, production status and genotype. The variance attributed to pre-analytical factors was also minimized by one normalization step before peak selection, and not two as proposed by Poon (2007), due to the risk of introducing “false” differences between profiles by this renormalization [11,19,27,50]. The difficulty in identification of proteins that correspond to the SELDI peaks is, as mentioned earlier, another major limiting factor, as also mentioned by Batxelli-Molina *et al*. and much effort should be made to identify these discriminating proteins, especially those which are significantly different between the groups [11].

## Conclusion

In conclusion, on the basis of the experimental infection model used, including route of infection and PrP genotype of the animals, we believe that the results in this study are relevant to the study of several aspects of naturally infected classical scrapie cases. Choosing peaks/proteins in biomarker research based solely on p-values from univariate models may, however, result in a number of false markers, and latent variable methods are much more suitable for these types of data. Such methods are simple to use for non-statistical users, and interpretation is made easy as results are visually well presented. This article describes one approach, from animal model to data analysis, and the resulting selection of significant protein peaks and creation of a predictive model. The results show that it is possible to use data from SELDI-TOF-MS in combination with multivariate data analysis to discriminate scrapie affected sheep from healthy controls. We identified one peak, or one discriminating protein, to be serum amyloid A (SAA), in the scrapie affected animals at the end stage. However, the practical application of this predictive model is restricted due to the limiting factors of SELDI-TOF MS. The multiple detected differences between these groups might, therefore, have been more completely illustrated by other -omic methods. Studies on differences in proteomic profiles between healthy and scrapie infected sheep will, undoubtedly, provide novel insight into the underlying pathogenic and pathological events. However, as long as these discriminating protein peaks remain unidentified, the pathological and clinical relevance of the actual proteins in relation to scrapie remains unknown. Our conclusion is therefore that there is a need for sensitive and specific bioassays using identified biomarkers, obtained by –omic methods, which can be utilized by various research groups across experiments.

## Materials and methods

### Animals

A total of 19 lambs over two consecutive years (2006 and 2007) were included in this study, all having the same PrP genotype, homozygous V_136_R_154_Q_171_ (Table 6). Lambs were inoculated orally with 1 gram homogenated pooled brain material from either healthy sheep or confirmed cases of classical scrapie immediately after birth and before any ingestion of colostrum and then grouped (control or scrapie group) according to inoculation material. Inoculation brain material used in both groups was tested for PrP^Sc^ by WB (Figure 1). The lambs were left with their mothers in confined isolated boxes under similar conditions and feeding regimes. All the lambs used were born within a time period of 15 days. At post mortem examination, the obex area of the brain from each animal was sampled for detection of PrP^Sc^ by WB (Figure 1). Animal experiments were approved by the Norwegian Animal Research Authority.

**Table 6:** Overview over samples, animals, genotype and age of sampling at end stage of disease

**Sample ID**	**Year**	**Group**	**Genotype**	**Sex**	**Age in weeks**
1, 2, 10, 11, 19	2006	Control	VRQ/VRQ	Male and Female	24 – 25
5, 13, 14, 22	2007	Control	VRQ/VRQ	Male and Female	25
3, 4, 12, 20, 21	2006	Scrapie	VRQ/VRQ	Male and Female	23
6, 7, 15, 23, 24	2007	Scrapie	VRQ/VRQ	Male and Female	23 – 24

### Serum samples

Serum samples used in this work were drawn every two weeks from six weeks post infection (p.i.) until euthanasia in 2007 for the longitudinal study (LS). Serum samples at time of euthanasia from both 2006 and 2007 were used for the end-stage study (ES). Serum samples were allowed to clot at room temperature for a minimum of 30 minutes and maximum 60 minutes, and then processed. Serum was pipetted in aliquots and frozen at minus 80 degrees within two hours of sampling. All the samples were subjected to the same handling procedures throughout the experiment.

### Serum fractionation

Serum samples were fractionated prior to SELDI-TOF MS analysis, using strong anion exchange fractionation kit, ProteinChip® Q Spin Columns (Bio-Rad), containing Q ceramic HyperD F sorbent. Before application to columns, proteins were denatured by addition of 150 μl 9 M Urea 2% Chapters 50 mM Tris–HCl pH 9 (U9) buffer to each of the 100 μl of serum samples, this followed by an additional 250 μl 1 M Urea 0,2% Chapters 50 mM Tris–HCl pH 9 (U1) buffer. The 500 μl serum mixture was added to the columns, and incubation time was set to 30 minutes at 4 degrees on a rotator to ensure complete mixing of serum mixture and column sorbent. Each sample was fractionated into six fractions (FT/F1, F2, F3, F4, F5 and F6). Flow through (FT) fraction was captured directly after sample incubation, and the consecutive fractions were captured after adding washing buffers with decreasing pH, starting at pH 9 and ending at pH 3 when capturing F5. The last fraction, F6, was captured after a wash with an organic buffer. The different fractions were aliquoted, and stored at – 80°C soon after capture until further analysis.

### SELDI-TOF MS analysis

A Weak cation exchange array (ProteinChip® CM10 Array, Bio-Rad) in combination with high stringency buffer, 50 mM HEPES pH 7.0 as binding and washing buffer was used to analyse the flow through (FT) fraction in this work. Each FT fraction was diluted 1:10 with binding buffer before application to array, and each individual LS and ES sample was applied randomly onto the array in three and five replicates, respectively. The matrix, ProteinChip® Sinapinic Acid (SPA) Energy Absorbing Molecules (EAM), was applied before the SELDI-TOF-MS analysis. The arrays were prepared and handled according to manufacturer’s instructions. The arrays were analysed on the Protein Biology System II (PBS-IIc) with autoloader (Bio-Rad Laboratories) using Ciphergen ProteinChip® Software Version 3.2.1. (ProteinChip® Software) with the integrated Biomarker WizardTM (BW) cluster analyses software [51]. Each chip was analysed with a spot protocol optimized for the low mass area (LM) between 2 and 25 kDa, and spectra were collected using an average of 130 laser shots. ES and LS samples were prepared and analysed separately. The BW feature of the ProteinChip® Software was used for peak clustering in the range of interest (2 kDa – 25 kDa).

### Data processing

Spectral data was processed to reduce instrumental and handling artefacts, minimize variation within groups and maximize variation between groups, and improve peak detection. Spectra were named and organised into groups according to age at sampling and group belonging (control and scrapie). Data were processed using ProteinChip® Software [51]. This process involved four steps; calibration, baseline subtraction, filtering and noise reduction and normalization (TIC). Finally, peak selection was performed by BW. Data processing was performed following recommendations described by Bio-Rad [36]. The collected peak data was exported into Microsoft® Office Excel 2003 and Sirius Version 8.1 (Pattern Recognition System AS, Bergen, Norway) for further data analyses. The spectra were evaluated for intra-cassette and inter-cassette reproducibility by calculation of the coefficient of variation (CV) for both peak intensity and peak mass (*m/z*). The CV for ES data set was calculated for each of the samples based on peak information in each of the five replicates, and CV for LS data set was calculated from peak information in a quality control (QC) sample that was repeatedly run with the samples.

A calibration equation was created using the calibration feature in the ProteinChip® Software and standards containing peptides and proteins of known mass (ProteinChip All-In-One Peptide/Protein Standard, Bio-Rad), which were run parallel to the samples. One equation for each data set, ES and LS, was calculated and applied to all the spectra in each of the respective study.

The shape of the baseline of each spectrum was examined and the baseline feature was used to subtract baseline. Fitting width was set to two times (2×) expected peak width. The noise range was set to 2 kDa to exclude matrix attenuation range from the analysis, and end was set to 100% of spectrum size.

The baseline and noise reduced spectra were normalized using the Total Ion Count (TIC) Normalization feature in the ProteinChip® Software, which normalizes each spectrum to equal sum detected signal under the curve in the region of interest. Each group, based on age and group belonging was normalized separately. The resulting normalization factor created for each spectrum was inspected and evaluated. Spectra with normalization factor above mean + 2 standard deviations were excluded from further analysis.

Peak clusters were generated using the BW function in the ProteinChip® Software to detect peaks of similar mass across the spectra. Peaks were detected using the following settings; first-pass detection with signal-to-noise ratio > 5, with cluster completion using a second-pass with signal-to-noise ratio > 2. The peaks needed to be present in at least 20% of the spectra (giving a presence in at least half of each group). A mass difference of 0.3% was allowed. Peak cluster information was exported to Excel for further analysis.

### Data analysis

#### Univariate

The data were tested for difference in relative peak intensity between the two groups using the non-parametric Mann–Whitney U test included in the BW and Sirius software. The fold change in intensity was calculated as the mean peak intensity control/mean peak intensity scrapie for significantly down-regulated peaks, and vice-versa for up-regulated peaks. For all tests, the significance level was set to p < 0.05.

#### Multivariate

Latent variable projection methods (LV) were used to analyse the SELDI-TOF-MS data. Both ES and LS data was analysed by principal component analysis (PCA) to visually evaluate the distribution of the data irrespectively of group belonging. Only ES data were further analysed using other LV methods. A group membership variable was defined, assigning “0” to all the samples in the control group, and “1” to all the members in the scrapie group. Partial least squares – Discriminant Analysis (PLS-DA) and target projection method (TP) were then used to evaluate the data distribution according to group membership. For all analyses, the spectral variables were standardized to unit variance, thereby preventing variables with high variance to dominate the data analysis. A non-parametric Discriminating Variable test (DIVA) was used to connect Selectivity Ratio (SR) value to the discriminatory ability of the variables, quantified as the probability of correct classification. Each variable got a correct classification rate (CR), i.e. how well each variable separated the two groups in question. The SR value was plotted against the Mean Wilcoxon Rank Sum Rate to obtain the DIVA plot.

Cross validation was used for ES data to optimize the LV models with respect to predictive performance. Different procedures for cross validation have been developed [52]. The ES data were split into four groups, constructing one PLS model for each group, one group was used as validation set and the others as training sets. The number of PLS components was chosen as the one giving the first minimum in prediction error.

### Protein identification

One ES sample from each of the groups was prepared and processed for protein identification. Thirteen μl of the FT fraction were mixed with 6 μl 4× LDS, 2.5 μl 10× DTT. The sample mixture was heated to 60°C for 15 minutes. 2.5 μl IAA (60 mM) was added to the mix and let to incubate for 15 minutes at room temperature and in the dark before loading on a 16% ClearPAGE gel (C.B.S. Scientific, USA). The gel was run at 150 V for 85 minutes. After electrophoresis the gel was stained with Gelcode Blue Safe Stain (Pierce, USA) for 1 hour and de-stained overnight with ultrapure water. Three protein bands in the region of 9 and 14 kDa bands on the gel were excised and subjected to tryptic digestion using OMX tube devices (OMX, Germany) following the manufacturer’s protocol.

Tryptic peptide samples were sent to International Research Institute in Stavanger (IRIS), Mekjarvik, Norway, and protein identification was done according their standard operating procedure. The protein identification was performed by LC-MS/MS analysis using an UltiMate 3000 dual pump nanoflow HPLC system (Dionex, Sunnyvale, CA, USA) connected to a linear ion trap-Orbitrap mass spectrometer (LTQ-Orbitrap XL, Thermo Fisher Scientific, Waltham, MA, USA). A sample volume of 5 μl from each sample was loaded onto a trapping column (Acclaim PepMap100 C18, 5 μm, 300 μm I.D. × 5 mm length, Dionex) at a flow rate of 2 μl/min in 0.1% formic acid (VWR) in MilliQ water (Elga) for clean-up and pre-concentration. Peptides were separated in the analytical column (Acclaim PepMap100 C18, 3 μm, 75 μm I.D. × 15 cm length, Dionex). The mobile phases for the analytical separation consisted of 0.1% formic acid in 2.5%/97.5% acetonitrile/water (A) and 0.1% formic acid in 80%/20% acetonitrile/water (B) and were pumped with a flow of 300 nL/min. The peptides were separated on the analytical column using a linear gradient from 5 to 60% B in 165 min after a 10 min delay post injection. The gradient was then run to 100% B in 10 min and held there for 30 min to wash the columns. A total run time of 256 min was used, including the washing step and 30 min re-equilibration of the columns. A PicoTip emitter (SilicaTip, New Objective) with a 10 μm tip and without coating was used as an ESI interface. The electrospray voltage was set to 1 kV, and no sheath gas was used. The mass spectrometer was used in positive mode. Full scans were performed in the Orbitrap in the *m/z* range from 200 to 2000, and data-dependent MS/MS scans performed in the linear ion trap for the five most abundant masses with z ≥ 2 and intensity ≥10000 counts. Dynamic exclusion was used with 3 min of exclusion after fragmentation of a given *m/z* value four times. Collision-induced dissociation (CID) was used with a collision energy of 35% and with activation Q setting of 0.400 and activation time of 30 ms for MS2. The mass spectrometer was tuned daily and calibrated weekly using the calibration solution recommended by Thermo Scientific.

Each LTQ-Orbitrap raw file was analysed using the Proteome Discoverer 1.0 (Thermo Fisher Scientific). Protein identifications were performed with the SEQUEST algorithm searching against even toed ungulate database available at NCBI with trypsin as digestion enzyme, and allowing for maximum two missed cleavage sites. Carbamidomethyl (C) was set as a static modification, and oxidation (M) as a dynamic modification. Precursor ion and fragment ion mass tolerances were set to 10 ppm and 0.8 Da, respectively. Results were filtered for minimum 2 peptides and using a high and medium significance XCorr Score adjusted for peptide charges (z), Table 7.

**Table 7:** High and medium confidence peptide filter settings

**Charge (z)**	**XCorr Score**	**XCorr Score**
	**High confidence**	**Medium confidence**
1	1.2	0.7
2	1.9	0.8
3	2.3	1.0
> = 4	2.6	1.2

## Abbreviations

TSEs: Transmissible spongiform encephalopathies; PrPC: Normal cellular prion protein; PrPSc: Scrapie prion protein; SELDI-TOF-MS: Surface Enhanced Laser Desorption/Ionization Time-of-Flight Mass Spectrometry; PCA: Principal component analysis; PLS-DA: Partial least square discriminant analysis; TP: Target projection; SR: Selectivity ratio; LC-MS/MS: Liquid chromatography tandem mass spectrometry; TIC: Total ion current; CV: Coefficient of variation; Da: Dalton; BW: Biomarker wizard; PC: Principal component; MWCR: Mean Wilcoxon classification rate; DIVA: Discriminating variable; z: Charge; LDS: Lithium dodecyl sulphate; DTT: Dithiothreitol; IAA: Iodine acetamide; LV: Latent variable; WB: Western blot; wpi: Weeks post inoculation.

## Competing interests

The authors declare that they have no competing interests.

## Authors’ contributions

SM carried out the proteomic studies, statistical data analysis, participated in protein identification and drafted the manuscript. OMK participated in the design of the study, statistical data analysis and helped to draft the manuscript. RA participated in the statistical data analysis. KB participated in the design of the study, carried out parts of the protein identification and helped to draft the manuscript. AH participated in the design and performance of the proteomic studies. MJU participated in its design and coordination and helped to draft the manuscript. All authors have read and approved the final manuscript.
